# A Case-Control Study of Risk Factors for Development of Type 2 Diabetes: Emphasis on Physical Activity

**DOI:** 10.2188/jea.12.424

**Published:** 2007-11-30

**Authors:** Luping Wang, Takuhiro Yamaguchi, Toshiko Yoshimine, Akane Katagiri, Kazuko Shirogane, Yasuo Ohashi

**Affiliations:** 1 Aventis Pharma Ltd.; 2 Department of Biostatistics, School of Health and Nursing Sciences, University of Tokyo.; 3 Department of Public and Occupational Health Nursing, University of Occupational and Environmental Health.; 4 Department of Pharmacoepidemiology, University of Tokyo.

**Keywords:** case-control studies, type 2 diabetes, dyslipidemia, physical activity

## Abstract

The aim of this case-control study was to evaluate the association between the lifestyle risk factors, especially physical activity, and the prevalence of type 2 diabetes and the comorbidity of type 2 diabetes and dyslipidemia in middle-aged Japanese urban population. Subjects (279 males and 119 females, 53.5±6.8 years old) were selected from one city office in Tokyo and consisted of type 2 diabetes cases (n=53), dyslipidemia cases (n=130), the comorbidity cases (n=58) and sex- and age-matched controls (n=155). A self-administered questionnaire was used to collect physical activity data using Baecke’s questionnaire translated and other lifestyle data. Our results revealed that physical activity was significantly associated with the reduction of the prevalence of type 2 diabetes and the comorbidity, and the sex- and age-adjusted odds ratios of the fourth quartile to the lowest one were 0.31 (95%CI:0. 12-0.81) and 0.32 (95%CI:0.13-0.81), respectively. Family history of diabetes and smoking were independent risk factors for the prevalence of type 2 diabetes and the comorbidity.

Diabetes mellitus is one of lifestyle-related diseases. Type 2 diabetes is the most common type of diabetes throughout the world, and is increasing dramatically in populations who undergo rapid Westernization of their lifestyle.^1^ In Japan, 85-95% of diabetes mellitus is type 2 diabetes. In this view, prevention against developing of type 2 diabetes is most valuable. Thus, studies of the risk factors that contribute to the development of type 2 diabetes are meaningful.

For the last decade, several prospective studies have documented a clear relationship between higher levels of physical activity and reduced incidence of type 2 diabetes.^2^^-^^7^ These associations were independent from obesity, alcohol consumption, lipoprotein concentrations, and other factors that predicted the increase of risk of type 2 diabetes.

Further evidence that physical activity might avert type 2 diabetes has come from a retrospective study,^8^ which generally showed that patients with diabetes reported less physical activity than those free of the disease. This was shown in multiple age-, race-, and sex-specific groups. Aside from above studies, an intervention trial among Swedish men with type 2 diabetes showed benefit from increased levels of physical activity.^9^

It is estimated that approximately 40% of patients with type 2 diabetes complicate dyslipidemia (the comorbidity of type 2 diabetes and dyslipidemia). Type 2 diabetes itself may induce diabetic dyslipidemia that manifests as hypertriglyceridaemia, hypercholesterol and low high-density lipoprotein (HDL) cholesterol. Conversely, dyslipidemia is an independent risk factor of the development of type 2 diabetes.^10^ Furthermore, type 2 diabetes and dyslipidemia are two components of the insulin-resistance syndrome of obesity, hypertension, glucose intolerance, and dyslipidemia, which co-relate to insulin resistance and hyper-insulinaemia.^11^ Patients with the comorbidity of type 2 diabetes and dyslipidemia have a higher risk to develop cardiovascular disease than those with either type 2 diabetes or dyslipidemia. Therefore, studies of risk factors contributing to the development of the comorbidity of type 2 diabetes and dyslipidemia are important.

To our knowledge, there had been no epidemiologic study using the same population to investigate simultaneously the risk factors of type 2 diabetes, and risk factors for the comorbidity of type 2 diabetes and dyslipidemia. In addition, reports on the effect of physical activity in detail upon these diseases are rare in Japan. Here, we planed to investigate risk factors of type 2 diabetes, dyslipidemia, and their comorbidity. The aim of this study was to evaluate the association between the lifestyle risk factors, especially the physical activity, and the prevalence of type 2 diabetes, and the comorbidity of type 2 diabetes and dyslipidemia.

## METHODS

### Subjects

This frequency matched case-control study, conducted in November 1999 in Tokyo, Japan, was composed of 3 case groups and a co-control group. The study population consisted of 3162 individuals aged from 35 years to 65 years at a city office. All of them should take a health examination every year. Three case groups were identified according to the following criteria: (1) Type 2 diabetes or impaired fasting glucose (IFG). The cases were those who had been diagnosed as type 2 diabetes based on medical records. Those with their casual plasma glucose concentration ≧200mg/dl or with fasting plasma glucose concentration ≧126mg/dl tested at this health examination were also identified as type 2 diabetes. Whereas, those with their fasting plasma glucose concentration between 110-125 mg/dl and without diabetes history were classified as IFG;^12^^,^^13^ (2) Dyslipidemia. These cases were those who had been diagnosed as hyperlipidemia based on medical records or questionnaire and/or were being treated with anti-cholesterol drugs. Those with their total cholesterol level ≧240mg/dl, and/or HDL cholesterol level ≦40mg/dl at this health examination were also considered to belong to this group; and (3) Comorbidity of type 2 diabetes and dyslipidemia. These cases who met both of (1) and (2) criteria were considered as the comorbidity. In addition, we identified a co-control group who did not meet all of the above criteria and was free of type 1 diabetes.

According to the above criteria, among this population, 98 cases that met criteria of type 2 diabetes or IFG, and 89 cases that met criteria of the comorbidity were all selected for this study. With frequency matching to the above 187 cases by age (age categories of 5-year) and sex, 196 cases were randomly selected from 658 subjects who met criteria of dyslipidemia, and 209 controls who met co-control group criteria were also randomly selected from 2,317 subjects by frequency matching.

### Data collection

We used medical records at the examination center to collect data on ID number, sex, age, height, weight, and blood tests. Special attention was paid to the events that happened before each subject’s reference date. The reference date for each subject was the day of diagnosis for the cases or that of the health examination. Questionnaires were mailed to 592 subjects and 400 responded, representing a response rate of 68%. The response rates among four groups were similar. Among these 400 subjects, four missed many items in the questionnaire and were excluded from the analysis, leaving 396 subjects for analysis.

### Questionnaire

The self-administered questionnaire contained seven pages and consisted of two parts: habitual physical activity and other lifestyle risk factors. The portion on habitual physical activity was a modified Baecke questionnaire in Japanese.^14^^,^^15^ The modified Baecke questionnaire in Japanese consisted of three sections: work activity, sports activity, and leisure activity excluding sports. Each section consisted of several questions scored on a five-point Likert scale, ranging from never to always or very often. For the two most frequently reported sports activities, additional questions inquired the number of months per year and hours per week of participation. This questionnaire (with translation available) was chosen because it was short, easy to fill in, and its validity and reproducibility were acceptable.^16^^,^^17^ However, the validity and reproducibility of translation version has not been performed in Japan.

The portion of questionnaire for other lifestyle risk factors was developed and modified based on other questionnaires.^18^ It was used to collect data on family history of diabetes, cigarette smoking, maximum body mass index (M-BMI), diet habits, and medical history including type 2 diabetes and dyslipidemia, as well as other personal information. Dietary habits included intake of meat, fiber, sweet, green vegetables, and others; each question has frequency scales, ranging from every day intake to rarely intake. Stress of life events included 18 items such as divorce, death of children or a spouse, family troubles, serious disease, and failure in certification examination.

### Assessment of physical activity and other factors

Subjects were assessed for habitual physical activities that included work activity, sports activity, and leisure activity excluding sports by Baecke’s physical activity index. We defined all physical activities as in the adulthood period and sport activity as regular sport or exercise performed for at least 1 year or more. More representative of lifetime occupation was ascertained to assess occupational physical activity. Three levels of occupational physical activity were defined: (1) The low level for occupations such as clerical work, specialist personnel, managerial personnel, driving, teaching, and medical practitioner. (2) The middle level for occupations such as safety foreman. (3) The high level for occupations such as construction work. Subjects who participated in regular sports reported type, frequency, duration, months per year and the number of years for sports. Sports were subdivided into three levels of physical activity according to tables prepared by the Health Promotion and Nutrition Division, Health Service Bureau, Ministry of Health and Welfare of Japan.^19^ If the answer to a question had a missing value, the third point of five-point Likert scale was imputed. We calculated approximate quartiles of Baecke’s physical activity index based on the distribution of all subjects in four groups who reported any activity. The lowest quartile served as the reference category.

The Maximum weight before each subject’s reference date was collected from the questionnaire. We also obtained data on weight at this examination, but did not use the values in the following risk factor analyses because of the possibility of post-diagnosed weight reduction. The M-BMI was calculated as maximum weight (kg)/height (m^2^). The subjects with M-BMI ≧25 according to the WHO criteria were categorized as overweight. Subjects were also divided into smokers and nonsmokers. The former were those who had been smoking or had stopped smoking within 5 years with a habit of smoking more than 5 cigarettes per day. The latter were those who had never smoked or had stopped smoking for at least 5 years. The length of the period without smoking was chosen according to recent reports that estimated the period of time necessary to eliminate most of the deleterious effect of smoking.^20^ Stress of life events was dichotomized as experience and non-experience. Diet habits were also dichotomized as daily intake and other. Family history of diabetes was defined as having one or both of parents with a history of diabetes.

### Statistical analysis

An odds ratio (OR) was used to estimate the relative risk of type 2 diabetes, dyslipidemia, and the comorbidity of both diseases according to the level of physical activity and other risk factors. Conditional logistic regression analyses were used to estimate the sex- and age-adjusted odds ratios and their 95% confidence intervals (CIs) taking account of blocks defined by sex and age groups. The multivariable-adjusted odds ratios were calculated by the conditional logistic regression including the following potential confounding factors in the models: parental history of diabetes, cigarette smoking, stress of life events, history of hypertension, overweight, and daily intake of meat, green vegetables and fiber. These factors were considered a priori to the established risk factors for type 2 diabetes and/or dyslipidemia, and included in all models regardless of statistical significance. For comparing the three case groups, the same confounding factors were commonly used in analysis of three case groups. The p values for the trend of physical activity were calculated by using the monotone score calculated from a median for each category of physical activity.

We also simultaneously assessed odds ratios of other lifestyle risk factors using the same conditional logistic regression models. The p values were two-sided and p values less than 0.05 were considered to be statistically significant. Statistical analyses were performed using the SAS software version 6.12.^21^

## RESULTS

The characteristics of these four groups at the health examination are shown in Table 1. The distribution of sex and age among four groups were similar because of the matching. The means of total cholesterol and HDL cholesterol levels were similar between the dyslipidemia and the comorbidity groups. In addition, there were no large difference in the means of plasma glucose levels between type 2 diabetes and the comorbidity groups.

**Table 1.** The characteristics of the study subjects.

Characteristic	Type 2 diabetes cases (n=53)	Dyslipidemia cases (n=130)	Comorbidity^¥^ cases (n=58)	Controls (n=155)
Sex				
Male	39	89	41	110
Female	14	41	17	45

Age (year)*	54.6 ± 6.7	53.7 ± 6.8	54.7 ± 5.8	52.5 ± 7.5

Blood data (mg/dl)*				
Total cholestrol	195.9 ± 25.7	225.5 ± 42.6	226.0 ± 45.3	193.7 ± 24.5
HDL cholestrol	61.1 ± 14.3	47.5 ± 18.4	46.1 ± 16.4	59.4 ± 12.0
Fasting plasma glucose (n)^#^	121.2 ± 16.3(24)	97.1 ± 12.4 (25)	124.6 ± 20.4 (24)	99.3 ± 12.4(11)
Causal plasma glucose (n)^#^	199.2 ± 109.1 (29)	97.7 ± 17.1 (104)	217.0 ± 119.2 (31)	98.1 ± 16.0 (138)

^#^ Calcutlated from participants who had blood test of this item.

^¥^ Comorbidity of type 2 diabetes and dyslipidemia.

* Mean ± standard deviation

Due to the small number of the cases with IFG (5 in total), we included these cases in type 2 diabetes. Thus, IFG cases were not assessed separately in the following analyses.

The physical activity, which is the focus of the present study, was estimated for all subjects included in the present study. The mean scores of physical activity indices for cases with type 2 diabetes, dyslipidemia and comorbidity of both diseases, and controls were 8.25, 8.54, 8.20, and 8.65, respectively.

### Physical activity

Table 2 shows odds ratios of cases with type 2 diabetes according to physical activity level. A total of 16 cases (32%) and 32 controls (22%) were categorized in the reference category, respectively. The sex- and age-adjusted OR of type 2 diabetes among subjects who reported physical activity level in the highest quartile was 0.31 (95% CI: 012-0.81), as compared to subjects who reported physical activity level in the lowest quartile. After adjustments for family history of diabetes, cigarette smoking, overweight, stress of life events, history of hypertension and daily intake of meat, green vegetables and fiber, increased physical activity was associated with a reduced risk of type 2 diabetes. The multivariable-adjusted OR among the highest quartile level was 0.18 (95% CI: 0.06-0.55) with the lowest quartile level as reference. The decline in the risk of type 2 diabetes with increasing physical activity level was statistically significant (p for trend in the sex and age-adjusted model was 0.017, and p for trend in the multivariable-adjusted model was 0.005).

**Table 2.** Odds ratios of type 2 diabetes according to physical activity level.

Physical activity level*	Cases (n=53)	Controls (n=155)	Sex- and age-adjusted odds ratio (95% CI)	Multivariable-adjusted odds ratio (95% CI)^#^
Lowest quartile	16	32	1.00	1.00
Quartile 2	16	38	0.76 (0.32-1.82)	0.43 (0.16-1.18)
Quartile 3	13	36	0.70 (0.28-1.71)	0.63 (0.23-1.72)
Highest quartile	8	49	0.31 (0.12-0.81)	0.18 (0.06-0.55)

p value for trend			0.017	0.005

* Quartiles (Baecke’s physical activity index): ≦7.6, 7.7-8.5, 8.6-9.3, and ≧9.4.

^#^ Adjusted for family history of diatetes, cigarette smoking, overweight, stress of life events, history of hypertension and meat, green vegetable and fiber intake.

CI: confidence interval

Among the cases with dyslipidemia and controls, 30 cases (27%) and 32 controls (25%) were used for the reference category (Table 3). There was no clear association between the risk of dyslipidemia and physical activity level. This result was drawn based on the ORs with adjustment for sex- and age-adjustment for the confounding factors mentioned above. The OR adjusted for all of the above confounding factors among the highest quartile level was 0.55 (95% CI: 0.26-1.16) in reference to the lowest quartile level. The monotonic dose-response relations were not statistically significant, (p for trend in the sex and age-adjusted model, and the multivariable-adjusted model were 0.202 and 0.167, respectively.)

**Table 3.** Odds ratios of dislipidemia according to physical activity level.

Physical activity level*	Cases (n=130)	Controls (n=155)	Sex- and age-adjusted odds ratio (95% CI)	Multivariable-adjusted odds ratio (95% CI)^#^
Lowest quartile	30	32	1.00	1.00
Quartile 2	34	38	0.89 (0.44-1.79)	0.70 (0.32-1.49)
Quartile 3	33	36	0.91 (0.46-1.84)	0.86 (0.40-1.82)
Highest quartile	33	49	0.65 (0.33-1.28)	0.55 (0.26-1.16)

p value for trend			0.202	0.167

* Quartiles (Baecke’s physical activity index): ≦7.6, 7.7-8.5, 8.6-9.3, and ≧9.4.

^#^ Adjusted for family history of diatetes, cigarette smoking, overweight, stress of life events, history of hypertension and meat, green vegetable and fiber intake.

CI: confidence interval

Table 4 shows the odds ratios of the comorbidity of both type 2 diabetes and dyslipidemia according to physical activity level. A total of 18 cases (33%) and 32 controls (22%) were categorized in the reference category, who reported physical activity level in the lowest quartile. Our results showed that there was an inverse association between the risk of the comorbidity and physical activity level. The sex and age-adjusted OR was 0.32 (95% CI: 0.13-0.81) for the highest quartile, as compared to the lowest quartile, which was used for reference. This relationship (i.e., the decline of risk of this comorbidity and increasing physical activity level) was significant (p for trend: 0.021). After adjustments for all the above confounding factors, the multivariable-adjusted OR was 0.36 (95% CI: 0.12-1.14). A monotonic dose-response relation was not statistically significant (p for trend: 0.134).

**Table 4.** Odds ratios of the comorbidity of type 2 diabetes and dislipidemia according to physical activity level.

Physical activity level*	Cases (n=58)	Controls (n=155)	Sex- and age-adjusted odds ratio (95% CI)	Multivariable-adjusted odds ratio (95% CI)^#^
Lowest quartile	18	32	1.00	1.00
Quartile 2	14	38	0.70 (0.30-1.68)	0.60 (0.20-1.78)
Quartile 3	17	36	0.81 (0.35-1.90)	0.91 (0.33-2.54)
Highest quartile	9	49	0.32 (0.13-0.81)	0.36 (0.12-1.14)

p value for trend			0.021	0.134

* Quartiles (Baecke’s physical activity index): ≦7.6, 7.7-8.5, 8.6-9.3, and ≧9.4.

^#^ Adjusted for family history of diatetes, cigarette smoking, overweight, stress of life events, history of hypertension and meat, green vegetable and fiber intake.

CI: confidence interval

### Other risk factors

According to the sex and age-adjusted ORs, family history of diabetes and cigarette smoking were found to be positively associated with the risk of type 2 diabetes. By contrast, daily intake of fiber was inversely associated with the risk of type 2 diabetes. After adjusting for physical activity level and other factors, the multivariable-adjusted ORs according to family history of diabetes, cigarette smoking, stress of life events and fiber intake were 5.24 (95% CI: 2.04-13.49), 2.50 (95% CI: 1.11-5.68), 4.50 (95% CI: 1.09-18.47), and 0.44 (95% CI: 0.19-0.99), respectively. Overweight, history of hypertension and daily intake of meat and vegetables were not shown to be significantly associated with the risk of type 2 diabetes.

Our results indicated that cigarette smoking and overweight were positively associated with the risk of dyslipidemia. After adjusted for the same the above confounding factors, the multivariable-adjusted ORs of cigarette smoking and overweight were 2.54 (95% CI: 1.43-4.52) and 1.98 (95% CI: 1.18-3.33), respectively. The risk of dyslipidemia in cases with smoking or overweight was almost two times higher than in controls that were nonsmoker or were not overweight. The others were not statistically significant.

These factors included family history of diabetes, cigarette smoking, overweight, history of hypertension were found to be positively associated with the risk of this comorbidity. The multivariable-adjusted ORs for family history of diabetes, cigarette smoking, overweight, history of hypertension were 3.95 (95% CI: 1.56-10.01), 3.30 (95% CI: 1.37-7.99), 3.36 (95% CI: 1.55-7.29), and 3.08 (95% CI: 1.24-7.63), respectively. The multivariable-adjusted ORs of other risk factors that were stress of life events, meat intake, green vegetable intake and fiber intake were not statistically significant.

## DISCUSSION

Results from our study suggest that physical activity as a protective effect is associated with a reduced risk of type 2 diabetes in middle-aged men and women. The protective effect is independent of family history of diabetes, cigarette smoking, overweight, stress of life events, history of hypertension, and daily intake of meat, green vegetables and fiber. However, an independent protective effect of physical activity (the multivariable-adjusted ORs) in the cases with the comorbidity was not statistically significant in the present study.

Although both of type 2 diabetes and dyslipidemia are lifestyle related diseases and co-related to insulin resistance and hyper-insulinaemia, the risk of dyslipidemia only was not significantly associated with physical activity level. This result was able to explain that there was no large difference in the protective effect of physical activity between type 2 diabetes group and the comorbidity group.

We also analyzed the associations between three case groups and work activity, sports activity and leisure activity excluding sports, respectively. The associations between three case groups and sports activity was similar to that mentioned above (physical activity included work activity, sports activity and leisure activity). However, there were not statistically significant associations between three case groups, and work and leisure activities. This might have been that the study population came from the city office and had similar office work and leisure activity.

Our results are consistent with several previous studies on the effect of physical activity upon the risk of type 2 diabetes. Three prospective cohort studies reported high levels of physical activity to be significantly associated with a reduced risk of type 2 diabetes.^6^^,^^8^^,^^22^ In one retrospective study, a reduced risk of diabetes was observed among women who engaged in regular sports in college than those who did not, but obesity was not adjusted in the analysis.^23^

The beneficial role of physical activity was not supported by previous studies. One prospective study^24^ reported no association between physical activity and the risk of type 2 diabetes. The discrepancy could be due to the study designs, subjects selected, and outcome measurement. In particular, a number of different methods to assess physical activity may have produced varying results. Although there may be some methodological differences, the majority of epidemiologic studies have shown an inverse association between physical activity and the risk of type 2 diabetes.

It is biologically plausible that physical activity might reduce the risk of type 2 diabetes, because physical activity increases glucose disposal through a number of pathways. Physical activity has independent effects on glucose disposal by increasing both of insulin-mediated and non-insulin-mediated glucose disposal.^25^ Recent evidence suggests that energy expended in physical activity increases peripheral sensitivity to insulin, especially in skeletal muscle and adipose tissue.^9^^,^^11^

It is known that insulin resistance is a pathogenesis of the comorbidity. In addition to effects mentioned above, physical activity induce plasma lipid and lipoprotein modifications, as well as the lipoprotein enzyme changes that related to a person’s apo E genotype.^26^

We also showed several independent risk factors for type 2 diabetes and/or the comorbidity: (1) Family history of diabetes: The high rate of type 2 diabetes among subjects with family history of diabetes is consistent with the results of previous studies.^11^^,^^27^ It is known that family history of diabetes operates through genetic mechanisms. The relative risk of type 2 diabetes was similar the comorbidity according to family history of diabetes; (2) Cigarette smoking: Our result is consistent with a previous study,^28^ in which cigarette smoking appears to markedly aggravate insulin resistance. The relative risk of type 2 diabetes and the comorbidity according to smoking was not remarkably different; (3) Stress of life events and less fiber intake are independent risk factors only for type 2 diabetes. The stress of life events is positively associated with the risk of type 2 diabetes through the increase of insulin resistance.^7^ The protective effect of daily fiber intake was found to agree with a previous report;^29^ (4) Overweight and history of hypertension are independent risk factors in subjects with the comorbidity. The results are supported by previous studies.^30^^,^^31^^,^^32^^,^^33^

In addition, smoking and overweight were found to be independent risk factors in the risk factors of dyslipidemia. By contrast, in all the three case groups, we did not find enough evidence that daily intake of meat has a positive association and daily intake of green vegetables has an inverse association with these diseases. These may be caused by the use of single questionnaires on diet habits that only included the frequency and did not include the amount of intake.

The pathogenesis for difference of relationship between risk factors, and type 2 diabetes only and the comorbidity has not been clear. By including dyslipidemia, the relationship between the risk factors and the comorbidity might have been diluted. Moreover, the relationship may be confounded by other risk factors or confounding factors.

There are several limitations in this study that should be considered when the results are interpreted. Firstly, information was collected on only physical activity in the adulthood period. The lack of exposure information regarding adolescent period may arise misclassification of physical activity. A recent publication provided the lifetime total physical activity questionnaire designed specifically for assessing relationship between physical activity and the risk of diabetes,^34^ but it is interviewer-administered questionnaire that is not practical in large population survey. Secondly, since subjects included diagnosed type 2 diabetes and diagnosed dyslipidemia, there was a possibility of recall bias in our study. This bias may give rise to underestimating or overestimating of the risk factors. Moreover, we must point out that the reference dates were the day of diagnosis for case and the day of the last examination for the control. Since the length and the calendar time of observation were different, it may also cause the bias for evaluating results. Thirdly, since all of our subjects were civil officers in Japan, it is not clear whether or not these findings can be generalized to other populations.
